# Effect of weekly Plyometric training frequency on Adolescents female basketball players during in-season: A comparison of two vs. four sessions

**DOI:** 10.1371/journal.pone.0320195

**Published:** 2025-04-28

**Authors:** Bruno Figueira, Eduardo Abade, Nuno Mateus, Anthony Weldon, Jaime Sampaio, Rūtenis Paulauskas

**Affiliations:** 1 Departamento de Desporto e Saúde, Escola de Saúde e Desenvolvimento Humano, Universidade de Évora, Évora, Portugal; 2 Comprehensive Health Research Centre (CHRC), Universidade de Évora, Évora, Portugal; 3 Educational Research Institute, Education Academy, Vytautas Magnus University, Kaunas, Lithuania; 4 Research Center in Sports Sciences, Health Sciences and Human Development, CIDESD, Elite Research Community, Vila Real, Portugal; 5 Department of Sports Science, Exercise and Health, School of Life Sciences and Environment, University of Trás-os-Montes and Alto Douro, Vila Real, Portugal; 6 Portugal Football School, Portuguese Football Federation, Oeiras, Portugal; 7 Department of Sports Sciences and Physical Education, University of Maia, Maia, Portugal; 8 Centre for Life and Sport Sciences, Birmingham City University, Birmingham, United Kingdom; 9 Aston Villa Foundation, Aston Villa Football Club, Birmingham, United Kingdom; University of Study of Bari Aldo Moro, ITALY

## Abstract

This study investigated the effects lower vs. higher frequencies of volume-equated plyometric training youth female basketball players. Thirty youth highly trained female basketball players (age, 15.7 ± 0.5 years; body mass, 64.1 ± 8.6 kg; height, 172.8 ± 6.2 cm, basketball training experience 6.3 ± 1.7 years) integrating a youth national development program participated in the study. A parallel-group randomized trial was undertaken to assess the effect of an eight-week plyometric intervention on jumping (counter-movement jump, drop-jump, horizontal jump), running (5 and 20-meter sprint), and change of direction performance. The study compared the outcomes of two versus four volume-equated training sessions per week, followed by a one-week retention period. A Bayesian Mixed Factor ANOVA revealed decisive evidence that the change of direction improved performance between the pre-test and post-test, as well as pre-test and retention. No discernible differences emerged between intervention groups. For the counter-movement jump, moderate evidence supported performance improvements in the 2PLYO group. In drop jump, both groups demonstrated decisive improvements between the pre-test and post-test, with moderate evidence for pre-test and retention, but no group differences were observed. These findings suggest that an 8-week plyometric training program, in both frequencies, leads to significant improvements in change-of-direction, countermovement jump, and drop jump performance among female junior basketball players participating in a youth national development program. However, it remains inconclusive whether a two-day training frequency provides a distinct advantage over four-days. Further research or consideration of additional factors may be necessary to ascertain the optimal training frequency for maximizing benefits.

## Introduction

Basketball requires players to perform brief short high-intensity efforts that require high levels of agility and power to achieve an advantage during a competition [1]. Previous research indicates that the proficiency in executing short, high-intensity actions, such as acceleration and jumps, correlates with game performance indicators in basketball [2] and should be consistently enhanced.

Plyometric training (PT) is a training method based on the stretch-shortening cycle (SSC), which involves a rapid transition between a stretch-intense eccentric action and a concentric shortening action [3]. The SSC utilizes the elastic properties of the muscle-tendon unit and the myotatic reflex, particularly when the ground contact time is less than 250 ms, enabling high-intensity force production and dynamic movements [3]. Plyometric exercises, such as jumping, hopping, and skipping, are effective in enhancing muscle fiber strength, contraction velocity, and the rate of force production, making PT a valuable tool for athletic performance development [4]. The effects of PT are well-documented, with research highlighting its ability to improve agility [5], coordination [6], acceleration [5], speed [7], power [8], muscle strength [9], and even muscle hypertrophy [10]. However, the effectiveness of PT is influenced by factors such as athletes’ characteristics, including age, gender, training background, sports activity, and prior experience with PT [11]. Training frequency also plays a critical role in determining PT’s impact on performance. Evidence suggests that a moderate frequency of two sessions per week yields better improvements in jumping performance compared to a higher frequency of four sessions per week [12]. This underscores the importance of balancing training volume and frequency to optimize adaptation stimuli and performance gains. Despite these findings, the lack of standardized training volumes across studies complicates the ability to draw definitive conclusions about the effects of distributing identical training volumes over varying frequencies [12]. While the optimal combination of program duration and training volume remains unclear, existing evidence suggests that extended session durations (>30 minutes), higher session frequencies (>two sessions per week), longer program durations (>eight weeks), and a high cumulative volume of sessions (>16) enhance the effectiveness of PT interventions [13]. Thus, this investigation is particularly relevant for teams facing constraints such as limited weekly training opportunities and the need to integrate a wide range of technical-tactical content.

The number of studies examining the effects of PT in female basketball players remains limited compared to those focusing on male athletes. Additionally, most existing research has involved small sample sizes and primarily targeted amateur teams [14,15], underscoring the need for more robust and comprehensive investigations to better understand PT’s effects in this population.

Effective training load management is crucial for optimizing athletic development and reducing the risk of overtraining and injuries, particularly in male youth team sport athletes, where workload variations align with different developmental priorities [16,17]. While recent meta-analyses have advanced our understanding of plyometric training frequency on performance outcomes [18,19], there is a lack of data examining the effects of manipulating training frequency while keeping overall volume constant. Exploring such strategies is essential, especially for enhancing horizontal and vertical explosive movements, which are key performance determinants in basketball.

While numerous studies have explored the effects of plyometric training on female basketball players, limited research has specifically investigated the impact of manipulating different PT frequencies while maintaining an equated total training volume on the overall physical performance of youth female basketball athletes. Of particular importance is that for basketball players the opportunity to engage in general athletic development during in-season period may be limited and require a compromise with short volume performance. To optimize the training plan, reducing the daily volume and increasing the weekly frequency can be an alternative to achieve better efficiency in exercises. Implementing 15–25-minute increments before basketball training enables the coach/athlete to effectively execute the general practice plan. Therefore, we hypothesize that a weekly equated PT load, distributed over four training sessions, may have a more pronounced impact on the jumping, running and change of direction speed of youth female basketball players compared to the same load distributed over two sessions.

## Materials and methods

### Participants

The sample size calculation was performed using G*Power software, version 3.1. Based on a Z1-β value of 1.03 (corresponding to a power of 85%) and a Z/2 value of 1.96 (indicating a significance level of 5%), the minimum required sample size for this study was determined to be 11 participants. This threshold aligns with the sample size used in a comparable study conducted by Gaamouri et. al., 2023 [20]. The participants were thirty highly trained youth female basketball players (age, 15.7 ± 0.5 years; body mass, 64.1 ± 8.6 kg; height, 172.8 ± 6.2 cm, basketball training experience 6.3 ± 1.7 years), familiarized with PT. The participants were recruited from a youth national development program, competing at youth national level league U-16, and supervised by Lithuanian basketball federation.

In brief, the inclusion criteria were: a) participate in a minimum of four basketball training sessions with at least 90-min duration; b) participate in one competitive game per week; and c) absence of musculoskeletal, neurological, or orthopaedic condition that may impede full participation in the exercise program.

### Procedure

Typically, training sessions had the following structure: warm-up consisting of low-intensity running, ball possession exercises, and dynamic stretching routines.; basketball drills focused on technical and tactical skills learning and improvement; small-sided basketball games; and 3x3 and 5x5 basketball games. Before the study, all participants underwent two individual strength training sessions per week, which focused on strength, speed, and flexibility training. The players were not involved in supervised strength training programs during the research period. The players, coaches, and legal guardians were fully informed about the study’s purpose, procedures, and risks and provided written informed consent before the study commenced. All athletes fully participated in every training session conducted over the entire duration of both interventions. The study protocol conformed to the recommendations of the Declaration of Helsinki and was approved and followed the guidelines stated by the Institutional Ethics Committee No. SA-EK-22–10. Before data collection, a computer-generated randomization schema was used to randomize participants (1:1) to a two-times-a-week (2PLYO, n = 15) or four-times-a-week (4PLYO, n = 15) PT group. (http://www.randomizer.org). Both groups performed the same weekly total volume of jumps during PT in addition to regular basketball training and competition. By selecting participants enrolled in the same youth national development program, we ensured that all athletes followed an identical training structure and workload, maintaining consistency in the typical microcycle throughout the intervention. The 2PLYO group performed 240 jumps over two sessions in non-consecutive days (120 jumps per session), and the 4PLYO group performed 240 jumps over four sessions (60 jumps per session). After the eight-week intervention, all players underwent a one-week retention period with no PT training while basketball training and competitions continued. Testing was performed at baseline (pre-test), after intervention (post-test), and after the retention (retention-test) period. Players were assessed for lower body power using jump tests (countermovement jump [CMJ], drop-jump [DJ] from a 20-cm box, and horizontal jump [HJ]; change of direction speed [CODS] test; and speed using a 20-m sprint and a 5-m split.

### Design

Players performed an eight-week plyometric intervention in addition to regular basketball practice and competition during in-season. One week before training and testing, all players participated in a familiarization session. The familiarization sessions began with a standardized 15-minute warm-up consisting of running, dynamic stretching, and ball possession drills to ensure participants were adequately prepared for subsequent activities. Following the warm-up, all exercises included in the PT protocol (see Table 1) were introduced by a certified strength and conditioning specialist. Each exercise was thoroughly demonstrated, and participants were instructed to perform two sets of each exercise under supervision to ensure proper technique and understanding of the protocol. The PT was performed at the beginning of basketball training sessions after a standardized warm-up routine consisting of highlight key exercises. The program aimed to develop key movement patterns and energy systems integral to the sport, such as jumping, acceleration/deceleration, and directional changes, while balancing horizontal (e.g., horizontal hops and bounds) and vertical force applications (e.g., repeated CMJ and jump and reach) alongside unilateral and bilateral exercises (e.g., double-leg tuck jump, split squat jump) [21]. The program was structured with a progressive load progression that began with low-impact, foundational plyometric tasks to establish neuromuscular control and movement proficiency. Over time, the exercises evolved into high-intensity, technically complex movements, ensuring a gradual yet robust increase in physical demands. This systematic approach facilitated safe and effective adaptations, emphasizing the enhancement of sport-specific strength, power, and movement efficiency [22]. Training drills were classified as low, medium, and medium-high intensity according to previous research [23]. The exercises performed, the number of repetitions, and the weekly progression for the PT are described in Table 1. All participants were monitored by experienced fitness coaches and continual instruction was provided to ensure safety, proper execution technique, and progression throughout the training.

**Table 1:** Plyometric Training Protocol.

Week	Intensity	Drill	2PLYO	4PLYO
			Sets x Reps (session)	Sets x Reps (session)
1-2	Low	Repeated CMJ	6x5	3x5
		Jump and reach	6x5	3x5
		Horizontal hops	6x5	3x5
		Lateral barrier hops	6x5	3x5
3-4	Medium	Single leg push-off	6x5	3x5
		Jump over barrier	6x5	3x5
		Double-Arm Alternated Leg Bound	6x5	3x5
		Lateral push-off	6x5	3x5
5-6	Medium	Double-leg tuck jump	6x5	3x5
		Split Squat jump	6x5	3x5
		Jump from box	6x5	3x5
		Side to side push off	6x5	3x5
7-8	Medium-high	Pike jump	6x5	3x5
		Front Barrier Hop	6x5	3x5
		Single-arm alternated bound	6x5	3x5
		Lateral box jump	6x5	3x5

Rest between sets: 0.5 min; rest between exercises: 2 min.

**Table 2:** Descriptive and reliability analysis of the player’s performance across assessment time-points.

Variables (group)	Pre-test		Post-test		Retention		ICC [95% CI]
	Mean ± SD	IQR	Mean ± SD	IQR	Mean ± SD	IQR	
**Horizontal jump (cm)**							
2PLYO	182.33±18.97	27.00	182.07±16.92	20.00	181.13±18.46	27.50	0.93 [0.85, 0.97]
4PLYO	187.60±17.42	20.00	191.33±18.32	21.50	188.71±15.02	13.50	0.88 [0.75, 0.96]
**CMJ (cm)**							
2PLYO	26.40±1.33	1.40	25.75±3.19	3.95	29.25±3.42	5.10	0.36 [0.05, 0.68]
4PLYO	24.29±4.44	4.40	26.07±3.98	4.59	24.40±2.68	3.71	0.89 [0.77, 0.96]
**Drop-jump (cm)**							
2PLYO	30.16±4.09	5.20	31.73±4.57	5.30	32.12±5.89	8.66	0.77 [0.55, 0.91]
4PLYO	28.82±3.28	2.75	30.24±3.53	4.30	31.77±2.16	2.84	0.68 [0.42, 0.87]
**Drop-jump RSI (a.u.)**							
2PLYO	1.46±0.38	0.51	1.43±0.28	0.42	1.55±0.36	0.56	0.45 [0.14, 0.74]
4PLYO	1.79±0.57	0.82	1.67±0.52	0.76	1.52±0.34	0.55	0.62 [0.34, 0.84]
**Linear sprint-5m (sec)**							
2PLYO	1.17±0.06	0.09	1.18±0.07	0.11	1.19±0.08	0.11	0.66 [0.39, 0.86]
4PLYO	1.12±0.09	0.12	1.16±0.09	0.11	1.16±0.08	0.10	0.47 [0.18, 0.75]
**Linear sprint-20m (sec)**							
2PLYO	3.56±0.18	0.24	3.59±0.17	0.29	3.59±0.16	0.21	0.86 [0.71, 0.95]
4PLYO	3.53±0.19	0.34	3.46±0.21	0.30	3.53±0.25	0.40	0.79 [0.58, 0.91]
**Change of direction (sec)**							
2PLYO	8.31±0.37	0.48	7.35±0.39	0.58	7.37±0.53	0.58	0.16 [-0.21, 0.53]
4PLYO	7.87±0.24	0.36	7.67±0.44	0.69	7.60±0.48	0.64	0.78 [0.56, 0.91]

Abbreviations: IQR = interquartile range; ICC = intraclass correlation coefficient.

The players underwent testing at baseline (pre-test), after an eight-week intervention (post-test), and after the one-week retention period. All the tests were accomplished in a single session between 6:00–8:00 p.m. on an indoor basketball court. One week before testing, familiarization with testing equipment and procedures took place. On testing days, participants completed a general warm-up that consisted of low-intensity running, dynamic stretching, body mass lower limbs exercises, and three 20-m sprints performed at 1/3 pace, 2/3 pace, and full pace, respectively. The players were required not to perform strenuous exercise 24 hours before testing, drink ad libitum and eating at least 2 hours before measurements.

### Outcome

#### Jumping performance.

Players performed two jumps for each protocol (CMJ, HJ, DJ - centimeters), with one minute rest provided between trials and three minutes rest between protocols. The best result was recorded for analysis. The CMJ and DJ height were calculated with an Optojump system (Microgate, Bolzano, Italy). The CMJ starts from a standing position with the hands on the hips. Then the player made a primary countermovement to their preferred depth, followed by an immediate maximal vertical jump effort. In the DJ, the players drop from a height of 20 cm with one foot, landing with 2 feet simultaneously, and then immediately perform a maximal jump. Flight and contact times recorded during a drop jump are utilized to calculate the RSI. The RSI serves as a key metric to evaluate stretch-shortening cycle efficiency and to determine optimal drop heights for maximizing the effectiveness of drop-jump training [24]. The RSI was calculated by dividing flight time (ms) by contact time (ms) [24]. In the HJ, a metric tape was used to measure the length between the players’ starting position and the nearest point of landing contact (i.e., the back of the heels). The players begin standing with their toes positioned against a marker. Players initiated the jump with countermovement and arm swing, jumping horizontally as far as possible and landing on both feet. The jump was invalid if the players did not land properly on their feet or fell back.

#### CODS.

The players performed CODS test (seconds) to measure the change of direction ability [23]. The players performed two trials separated by three minutes of rest. The fastest of the two trials was used for analysis. The performance time was measured using electronic photocells (Timing-Radio Controlled; TTSport, San Marino, CA, USA) [25], poisoned 3 meters apart and located on either side of the start and finish lines. All photocells were mounted at a height of 1 m above the floor, the maximum height of the manufacturer’s standard tripods. The players started the trials from a line placed 0.3-m behind the start line. Participants performed two 20-m sprints (with 5-m split time also recorded), with at least three minutes of rest between them [26]. The fastest performance trial was used for data analysis. During the recovery period between sprints, the participants returned to the starting line and waited for the second trial. Sprint times were recorded with electronic photocells (Timing-Radio Controlled; TTSport, San Marino, CA, USA) placed 5-m- and 20-m from the starting line. Players were instructed to start the sprints from a line set 0.3-m behind the start line, responding to an external stimulus, and to run at maximum effort through the final pair of sensors. During all trials of all tests, players were strongly verbally encouraged to produce their maximal effort.

### Statistical analysis

A boxplot and a Shapiro-Wilk test were used in all data sets to identify outliers and test distribution. Extreme outliers were removed to ensure data quality and mitigate undue influence on group-level analyses. Afterward, a Bayesian Mixed Factor ANOVA was used to quantify the interaction between the group and the time-point on the performance variables. The performance variables were used as the dependent variable, and the time-point and the groups were designated as repeated measures and between-subjects factors, respectively [27]. If the Bayesian Mixed Factor ANOVA yielded meaningful predictors (i.e., models outperforming the null model), post hoc tests were performed to determine time-point and group differences. Thresholds for Bayes factors were: < 0.01, decisive evidence in favor of the null hypothesis; 0.01–0.03, very strong evidence in favor of the null hypothesis; 0.03–0.1, strong evidence in favor of the null hypothesis; 0.1–0.3, moderate evidence in favor of the null hypothesis; 0.3–1, anecdotal evidence in favor of the null hypothesis; 1, no evidence; 1–3, anecdotal evidence in favor of the alternative hypothesis; 3–10, moderate evidence in favor of the alternative hypothesis; 10–30, strong evidence in favor of the alternative hypothesis; 30–100, very strong evidence in favor of the alternative hypothesis; > 100, decisive evidence in favor of the alternative hypothesis (44). Finally, the Cauchy prior width was set at r-scale fixed effects = 0.5 [27]. All testing calculations were performed using JASP software (JASP Team 2019. JASP for Windows, Version 0.19.0.0, computer software, Amsterdam, Netherlands).

## Results

**Fig 1:**
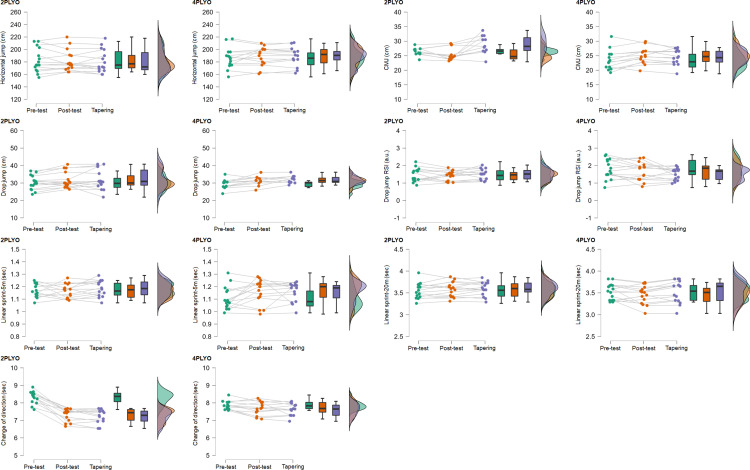
Raincloud plots show the distribution of the performance variables according to the plyometric groups. The clouds of points indicate all data points, the boxplots indicate the median and the first quartile (25^th^ percentile), and the third quartile (75^th^ percentile), and the one-sided violin plots indicate the data distribution for each performance measure.

Table 2 summarizes the descriptive statistics (mean, standard deviation (SD), and interquartile range (IQR)) and reliability measures (intraclass correlation coefficients (ICC) with 95% confidence intervals) for both groups (2PLYO and 4PLYO) across the pre-test, post-test, and retention assessments. Additionally, Fig 1 illustrates individual performance trajectories and data distributions for all variables across these time points.

Tables 3–5 show the inferences of the Bayesian Mixed Factor ANOVA and post hoc comparisons. The Bayes factor (BF_10_) indicated decisive evidence that the change-of-direction data is best represented by a model that included the time point, the group, and the interaction between the time point and the group as the predictor (BF_10_ = 1.19*10^16^). Post hoc comparisons revealed decisive differences between the pre-test × post-test (posterior odds = 3465.38) and between the pre-test × retention (posterior odds = 22032.26). Conversely, post-test × retention (posterior odds = 0.32) and group comparisons (posterior odds = 0.36) showed anecdotal evidence in favor of the null hypothesis (i.e., no differences). Similarly, the BF_10_ indicates decisive evidence (BF_10_ = 518.20) that the CMJ data is best represented by a model that includes the time point, the group, and the interaction between the time point and the group as the predictor. Post hoc comparisons of post-test × retention (posterior odds = 0.59) and pre-test × post-test (posterior odds = 0.18) revealed no differences. Additionally, post hoc comparisons exposed moderate differences between pre-test × retention (posterior odds = 3.38) and decisive differences between groups (posterior odds = 109.44).

**Table 3:** Model comparison for the Mixed Factor Bayesian ANOVA, considering the time-point and group factors.

Models	P (M|data)	BF_M_	BF_10_	Error %
*Horizontal jump (cm)*				
Null model	0.29	1.66	1.00	
Group	0.62	6.65	2.13	59.89
Time Point	0.04	0.16	0.13	0.89
Time Point + Group	0.04	0.14	0.12	13.25
Time Point + Group + Time Point * Group	0.01	0.04	0.03	3.49
*CMJ (cm)*				
Null model	0.00	0.01	1.00	
Time Point + Group + Time Point * Group	0.95	83.66	518.20	7.29
Time Point + Group	0.03	0.12	15.86	3.96
Group	0.01	0.03	4.38	1.19
Time Point	0.01	0.03	3.54	0.68
*Drop-jump (cm)*				
Null model	0.01	0.05	1.00	
Time Point	0.60	6.03	54.62	0.73
Time Point + Group	0.32	1.89	29.22	2.39
Time Point + Group + Time Point * Group	0.06	0.26	5.53	3.31
Group	0.01	0.02	0.51	1.83
*Drop-jump RSI (a.u.)*				
Null model	0.35	2.19	1.00	
Group	0.29	1.59	0.81	1.26
Time Point	0.13	0.61	0.37	1.49
Time Point + Group + Time Point * Group	0.12	0.53	0.33	5.69
Time Point + Group	0.11	0.52	0.32	5.58

All models include subject. All models were compared to the null model. The prior model probabilities were all equal (0.2). Abbreviations: P (M|data) = posterior model probability; BF_M_ = posterior model odds; BF_10_ = Bayes factor; RSI = reactive strength index.

**Table 4:** Model comparison for the Mixed Factor Bayesian ANOVA, considering the time-point and group factors.

Models	P (M|data)	BF_M_	BF_10_	Error %
*Linear sprint-5m (sec)*				
Null model	0.22	1.09	1.00	
Time Point	0.26	1.38	1.19	0.86
Time Point + Group	0.19	0.94	0.89	1.17
Time Point + Group + Time Point * Group	0.18	0.88	0.84	1.46
Group	0.16	0.75	0.73	0.96
*Linear sprint-20m (sec)*				
Null model	0.39	2.56	1.00	3.54
Group	0.29	1.59	0.73	1.24
Time Point	0.14	0.63	0.35	2.05
Time Point + Group	0.09	0.43	0.25	3.05
Time Point + Group + Time Point * Group	0.09	0.41	0.24	3.54
*Change of direction (sec)*				
Null model	8.43*10^-17^	3.37*10^-16^	1.000	
Time Point + Group + Time Point * Group	1.00	1.95*10^8^	1.19*10^16^	1.40
Time Point	1.39*10^-8^	5.54*10^-8^	1.64*10^8^	0.93
Time Point + Group	6.66*10^-9^	2.67*10^-8^	7.91*10^7^	1.02
Group	3.29*10^-17^	1.32*10^-16^	0.39	1.26

All models include subject. All models were compared to the null model. The prior model probabilities were all equal (0.2). Abbreviations: P (M|data) = posterior model probability; BF_M_ = posterior model odds; BF_10_ = Bayes factor.

**Table 5:** Post hoc comparisons, considering the time-point and group.

Models	Prior Odds	Posterior Odds	BF_10, U_	error %
*Horizontal jump (cm)*				
Pre-test × Post-test	0.59	0.14	0.24	0.00
Pre-test × Retention	0.59	0.13	0.22	0.00
Post-test × Retention	0.59	0.13	0.21	0.00
2PLYO × 4PLYO	1.00	0.74	0.74	0.02
*CMJ (cm)*				
Pre-test × Post-test	0.59	0.18	0.31	0.04
Pre-test × Retention	0.59	3.38	5.75	0.00
Post-test × Retention	0.59	0.59	1.01	0.00
2PLYO × 4PLYO	1.00	109.44	109.44	0.00
*Drop-jump (cm)*				
Pre-test × Post-test	0.59	332.67	566.35	0.00
Pre-test × Retention	0.59	6.12	10.42	0.00
Post-test × Retention	0.59	0.16	0.28	0.03
2PLYO × 4PLYO	1.00	0.27	0.27	0.02
*Drop-jump RSI (a.u.)*				
Pre-test × Post-test	0.59	0.26	0.45	0.02
Pre-test × Retention	0.59	0.30	0.52	0.02
Post-test × Retention	0.59	0.15	0.25	0.00
2PLYO × 4PLYO	1.00	1.51	1.51	0.00
*Linear sprint-5m (sec)*				
Pre-test × Post-test	0.59	0.41	0.69	0.00
Pre-test × Retention	0.59	1.30	2.21	0.00
Post-test × Retention	0.59	0.14	0.25	0.00
2PLYO × 4PLYO	1.00	1.18	1.18	0.01
*Linear sprint-20m (sec)*				
Pre-test × Post-test	0.59	0.16	0.27	0.00
Pre-test × Retention	0.59	0.15	0.25	0.00
Post-test × Retention	0.59	1.44	2.45	0.00
2PLYO × 4PLYO	1.00	0.91	0.91	0.00
*Change of direction (sec)*				
Pre-test × Post-test	0.59	3465.38	5899.51	0.00
Pre-test × Retention	0.59	22032.26	37508.04	0.00
Post-test × Retention	0.59	0.32	0.54	0.00
2PLYO × 4PLYO	1.00	0.36	0.36	0.02

The posterior odds have been corrected for multiple testing by fixing to 0.5 the prior probability that the null hypothesis holds across all comparisons [44]. Individual comparisons are based on the default t-test with a Cauchy (0, r = 1/sqrt (2)) prior. The “U” in the Bayes factor denotes that it is uncorrected. Abbreviations: RSI = reactive strength index.

Concerning the DJ, the Bayesian Mixed Factor ANOVA indicated very strong evidence that the data were 54.62 times more likely to occur under a model that included the time point as the predictor. Furthermore, post hoc comparisons of pre-test × post-test (posterior odds = 332.67) and pre-test × retention (posterior odds = 6.12) revealed decisive and moderate differences. In contrast, post-test × retention comparisons (posterior odds = 0.16) and group comparisons (posterior odds = 0.27) suggested no differences. Additionally, anecdotal evidence for the alternative hypothesis was observed for the 5-m linear sprint and the HJ performance (i.e., time point and the group model), whereas the BF_10_ indicates anecdotal evidence for the null hypothesis in the DJ RSI and the 20-m linear sprint.

## Discussion

This study examined the effects of distributing load-equated PT over two versus four training sessions per week during an eight-week intervention on the physical performance of female youth basketball players. The results indicated that both training approaches led to improvements in change of direction speed and DJ performance, which were sustained during the retention week. Regarding the CMJ, retention resulted in improved performance in the 2PLYO intervention. No differences were observed between testing moments and PT interventions for HJ performance, DJ RSI, and linear sprint (5-m and 20-m). This suggests that coaches have the flexibility to determine the frequency of PT sessions. Specifically, it is crucial to ensure that the training intensity on the day before a match and during intervals between consecutive matches is carefully managed, minimizing the risk of overloading athletes, thereby preserving performance capacity, and optimizing recovery.

In drop-jump (DJ), both groups showed a performance enhancement after the PT interventions. Further, players of both groups maintained performance improvements during the retention period, suggesting that volume and intensity may play a more critical role than training frequency in these adaptations. The beneficial effects of PT on jump height in different types of vertical jumps have been widely studied [32]. Vertical jump performance appears to take advantage of the subjects’ ability to use the elastic and neural benefits of the stretch-shortening cycle [13]. Meta-analytical studies in basketball associate strong reliance on vertical expressions of power when players are defending, shooting, and rebounding [33]. Improvements in vertical jump after PT may be attributed to various adaptive mechanisms, such as enhanced motor unit recruitment, greater inter-muscular coordination, heightened neural drive to agonist muscles, and enhanced utilization of the stretch-shortening cycle [34]. Additionally, both interventions also resulted in enhanced performance after one-week retention period. As previously mentioned, retention periods in which the regular training of a specific sport is maintained allow an athlete to maintain the gains previously achieved [35], which was confirmed for the jumping performance in our investigation. Indeed, it was already shown that jump performance may be maintained at a high level using sport-specific training only and that super-compensation can be achieved when low-load training phases are preceded by plyometric interventions [36].

Furthermore, the players in the 2PLYO group demonstrated improvement in CMJ performance between the pre-test and retention-test. Likewise, basketball involves several vertical intermittent high-intensity actions that may have potentiated the recovery between sessions and, consequently, CMJ results [37]. In addition, the tapering phase is a common strategy to reduce the physiological stress of training and increase performance [38]. Thus, the opposite results presented by both interventions can reflect that the retention period in 2PLYO was enough to promote super-compensation, usually observed a few days after the reduction of training volume, and positively associated with vertical jump capacity and sprint ability [38,39].

The specificity of PT is closely linked to load orientation exercise type of exercise, serving as primary determinants of training adaptations [40]. Acknowledging that the direction of PT activity, such as horizontal exercises, notably influences dynamic movements of the same orientation, like HJ [32], affirms the relevance of targeted training approaches. Despite sprint performance being influenced by both vertical and horizontal forces, the 20-meter sprint performance did not exhibit improvement in neither of the interventions. This limitation may be attributed to the inadequate inclusion of a variety of horizontally-oriented exercises, failing to elicit the requisite adaptations necessary for enhanced sprint performance and HJ [41].

CODS tasks require rapid transitions between eccentric and concentric muscle actions in the leg-extensor muscles, which are integral to the stretch-shortening cycle [3]. Evidence suggests that PT can enhance CODS performance by reducing ground-reaction times, primarily attributed to increased muscle-force output and greater movement efficiency [28]. However, the effects of PT are not uniform and may vary based on individual characteristics. Key factors as resistance training, muscle stretching, which are known to influence COD abilities, are heavily dependent on the specificity of the training regimen [4].Previous research has established that non-specific jumping exercises do not lead to improvements in CODS performance [29]. In contrast, incorporating specific training drills (e.g., lateral bounds, side hops, angles hops) has been shown to significantly enhance COD performance [30]. According to Asadi et al. [4], PT represents an effective strategy for substantially improving COD performance. These findings suggest that the benefits of PT extend beyond isolated improvements in movement efficiency, potentially transferring to enhanced athletic performance, particularly in sports such as basketball. Thus, including vertical, horizontal, and lateral plyometric drills in the present study might have played an essential role in improving COD performance in both groups. Under this scope, coaches may choose to condense or distribute the total number of jumps during the week according to their weekly training organization since both two and four PT interventions per week have similar positive effects for CODS that are maintained after a one-week unload period. The unchanged performance after the retention phase highlights the importance of PT, which is consistent with the nature of basketball training and competition [31]. The similarity in the results obtained by both groups seems to suggest that volume and intensity appear to have a greater influence on the outcomes than weekly frequency.

Despite sprint performance being influenced by a combination of vertical and horizontal forces, the lack of improvement in 20-m sprint performance could be attributed to an insufficient number or inappropriate selection of horizontal force-oriented exercises. These exercises are critical for acceleration and directional changes due to their role in enhancing forward propulsion and eccentric deceleration capacity [41]. Regularly performing sprinting bouts during defensive and offensive game situations in basketball has been shown to cause adaptive phenomena [2,33]. This may be due to increased neuromuscular activation during the game, resulting in improvements in sprint performance [33]. The observed improvements in 5m-linear sprint performance may be attributed to the number and firing frequencies of activated motor units, as well as changes in the recruitment pattern of the motor units (primarily in fast-twitch muscle fibers) [42]. These adaptations will likely increase maximal muscle force and power capabilities, enabling players to accelerate more rapidly at the start of sprints and execute longer stride lengths as sprints progress [43]. A meta-analysis by Ramirez-Campillo et al. [34] found that when PT involves a combination of horizontal and vertical jumps, horizontal force-related capabilities are particularly relevant in the acceleration phase of linear sprints (i.e., 10 m), whereas vertical force applied to the ground becomes more prominent as sprints progress and speed increases (i.e., >10 m). Therefore, a combination of horizontal and vertical jumps, appropriately distributed plyometric training during the microcycle, and the number of games during the micro-cycle may be essential for basketball players aiming to improve their sprinting performance. Furthermore, it is essential to emphasize the holistic importance of all training stimuli, as these collectively contribute to the athlete’s overall performance adaptations. Nevertheless, some limitations may be acknowledged. A key limitation of this study is its exclusive focus on players enrolled in a national development program, coupled with the relatively narrow age range of the participants. These factors restrict the generalizability of the findings to broader populations, including athletes from different developmental stages, competitive levels, or sports backgrounds.

## Conclusion

This study demonstrates that an 8-week load-equated PT intervention, delivered over two or four days significantly enhances physical performance in female junior basketball players.

Both 2PLYO and 4PLYO demonstrated improvements in the (DJ). In terms of the CMJ, 2PLYO displayed enhancements, while 4PLYO did not produce noticeable effects. Finally, CODS exhibited improvements in both training protocols. However, it cannot be conclusively argued that a two-day training frequency holds a significant advantage over four. Additionally, the improved DJ and CODS performance following the one-week retention period underscores the specificity of PT, aligning with the demands of basketball training and competition.

## Practical applications

The findings of this study highlight that coaches can tailor the frequency of PT sessions to align with effective training load management strategies. To optimize performance and recovery, it is essential to carefully regulate training intensity, particularly during congested schedules or high-demand periods. Furthermore, strategically manipulating training load frequency within the micro-cycle offers a practical approach to achieving performance enhancements while minimizing the risk of overtraining or injury.
